# Period, birth cohort and prevalence of dementia in mainland China, Hong Kong and Taiwan: a meta-analysis

**DOI:** 10.1002/gps.4148

**Published:** 2014-05-22

**Authors:** Yu-Tzu Wu, Hsin-yi Lee, Samuel Norton, A Matthew Prina, Jane Fleming, Fiona E Matthews, Carol Brayne

**Affiliations:** 1 Department of Public Health and Primary Care, Institute of Public Health, Forvie Site, University of Cambridge School of Clinical MedicineCambridge, UK; 2 Department of Health, Behavior and Society, Johns Hopkins Bloomberg School of Public HealthBaltimore, MD, USA; 3 Psychology Department, Institute of Psychiatry, King's College LondonLondon, UK; 4 Centre for Global Mental Health, Institute of Psychiatry, King's College LondonLondon, UK; 5 MRC Biostatistics Unit, Institute of Public Health, Forvie Site, University of Cambridge School of Clinical MedicineCambridge, UK

**Keywords:** prevalence of dementia, temporal variation, period, cohort effect, China, meta-analysis

## Abstract

**Objective:**

There have been dramatic societal changes in East Asia over the last hundred years. Several of the established risk factors could have important period and cohort effects. This study explores temporal variation of dementia prevalence in mainland China, Hong Kong and Taiwan taking study methods into account.

**Methods:**

Seventy prevalence studies of dementia in mainland China, Hong Kong and Taiwan were identified from 1980 to 2012. Five period groups (before 1990, 1990 ∼ 1994, 1995 ∼ 1999, 2000 ∼ 2004 and 2005 ∼ 2012) and five birth cohort groups (1895 ∼ 1909, 1910 ∼ 1919, 1920 ∼ 1929, 1930 ∼ 1939 and 1940 ∼ 1950) were categorised using the year of investigation and 5-year age groups. Pooled prevalence by age, period and birth cohort groups was estimated through meta-regression model and meta-analysis taking diagnostic criteria and age structure into account.

**Results:**

After adjusting for diagnostic criteria, the study age range and age structure, the prevalence of dementia in the older population aged 60 years and over fluctuated across periods but not reaching significance and were estimated as 1.8%, 2.5%, 2.1%, 2.4% and 3.1% for the five periods from pre-1990 to 2005 ∼ 2012. A potential increasing pattern from less to more recent birth cohort groups was found in the major studies using older diagnostic criteria with wider differences in the age groups over 70 years.

**Conclusions:**

This study found no significant variation across periods but suggested a potential cohort effect. The influence of societal changes might moderate early life experiences across different generations with substantial impact on mental health in older age. © 2014 The Authors. *International Journal of Geriatric Psychiatry* published by John Wiley & Sons Ltd.

## Introduction

Economic development and change in social environment affect the living conditions of people and exert substantial influences on the health of different generations (Wilkinson, 1992, 1997; WHO, 2008). Turbulence in the social environment can impact directly on a population's health and well-being (Shkolnikov *et al*., 2001; Men *et al*., 2003). The change of social environment is associated with several factors related to dementia, such as life expectancy, education opportunities, nutrition, stress and quality of life (Hendrie *et al*., 2006; Hall and Hendrie, 2012). These factors might have direct impact or lifetime influence on cognitive function in later life and cause varying prevalence of dementia across different periods.

Although the worldwide age-specific prevalence of dementia has generally been assumed to remain constant over the years in the report of Alzheimer's Disease International, change of dementia prevalence across periods in mainland China, Hong Kong and Taiwan has been reported by some reviews (Dong *et al*., 2007; Fuh and Wang, 2008; Yu *et al*., 2012; Zhang *et al*., 2012; Liu *et al*., 2013; Prince, 2013; Prince *et al*., 2013). A recent Lancet review that summarised prevalence, incidence and mortality studies of dementia in mainland China from 1990 to 2010 reported an increasing pattern of age-specific prevalence in the past 20 years (Chan *et al*., 2013). However, study methods and the assessment of cognitive frailty also change with time and could lead to the variation between different periods. Diagnostic criteria of dementia, age range, study sample size and sampling method have been shown to explain the varying results between prevalence studies in a further systematic review containing only population-based studies (Wu *et al*., 2013). Without controlling for the influence of methodological factors, temporal variations of dementia prevalence cannot be examined appropriately, and any claims of change in prevalence should be treated with caution.

### Societal changes and dementia

Dramatic societal changes in East Asia over the last hundred years could have caused in negative life experiences in early years with a major influence on health and quality of life across the life span and be associated with potential poor health outcomes in later life (Pensola and Martikainen, 2003; WHO, 2008). China, the largest country in this region, sustained an imperial system until the first decade of the 20th century. In transition to a modern society, China has undergone rapid changes of government and frequent wars, consequent chaos and political conflicts for over 50 years and then, more recently, striking economic growth (Chang, 1987). It is an immense, heterogeneous rapidly changing society whose people have experienced powerful and contradictory social forces (Kleiman, 1996). These critical changes and social forces would be expected to affect the living conditions of citizens and influence the health of populations with important effects on different birth cohorts.

Several studies have reported the potential association between economic development, societal changes and increasing prevalence of non-communicable diseases and common mental disorders, which are considered as important risk factors of cognitive decline and dementia in later life (Boutayeb and Boutayeb, 2005; Hendrie *et al*., 2006; Fu *et al*., 2013; Shao *et al*., 2013). However, the chaos in mainland China from 1950s to 1970s is considered to have had substantial impacts including disorganisation of the education system, lowered living conditions and the decreased life expectancy. Considering the influence of societal turbulence and high mortality, the prevalence of dementia in China is expected to be stable over these 20 years, but potential variation might be found in different generations who have various life experiences and living conditions.

This study builds on an earlier systematic review to explore the possible variation of dementia prevalence across different periods and birth cohort in mainland China, Hong Kong and Taiwan taking study methods into account (Wu *et al*., 2013). To the best of our knowledge, it is the first time that the temporal variation of dementia prevalence in mainland China, Hong Kong and Taiwan has been considered from both period and cohort perspectives with the exploration of the change of social environment on mental illness in older population.

## Method

### Literature search

A detailed literature search of prevalence studies in mainland China, Hong Kong and Taiwan conducted and reported in full elsewhere is summarised briefly here (Wu *et al*., 2013). An electronic search was conducted in three English (PubMed, ScienceDirect and PsycInfo) and two Chinese databases (Chinese National Knowledge Infrastructure and Airti Library) to identify the literature related to ‘prevalence/epidemiology’ and ‘dementia/Alzheimer’ in mainland China, Hong Kong and Taiwan (‘China/Chinese/Taiwan/Taiwanese‘) from 1980 to 2012. Both traditional and simplified characters were used to search the Chinese database. The following inclusion criteria were used to select papers: (i) cases were collected by field survey, not based on hospital data; (ii) the study involved population sampling rather than recruited volunteer participants; (iii) the study reported prevalence in the people aged 50 years and over; and (iv) dementia case was not decided only by a screening test and the specific instruments and criteria for case identification were reported. Studies were excluded if they were as follows: (i) duplicate; (ii) irrelevant or with other focuses (such as behavioural psychological symptoms of dementia and dependency in older populations); (iii) the results of follow-up waves; and (iv) focused on Chinese populations outside mainland China, Hong Kong and Taiwan.

Details of each study such as methodological factors (screening tools, diagnostic criteria and instruments), characteristics of the study population (sample size and response rate, the whole study age range and location) and results (overall prevalence of all types of dementia and stratified prevalence by age) were extracted systematically by two readers with double verification. Disagreements between the two readers were reconciled through discussion to arrive at a consensus.

### Period groups

Studies were categorised on the basis of beginning year of investigation (not publication year) into five groups: before 1990, 1990 ∼ 1994, 1995 ∼ 1999, 2000 ∼ 2004 and 2005 ∼ 2012 (Wu *et al*., 2013). If there was no clear information about year of investigation in the paper, publication year minus 3 years was used as an approximation for the survey date, because the median time between beginning year of investigation and publication year was 3 years in the rest of the studies.

### Age groups

Most of the studies reported prevalence of dementia by 5-year age groups. Because the three studies with the age range 50 years and above did not provide age-specific prevalence by 5 years, the following categories were used: 55–59, 60–64, 65–69, 70–74, 75–79, 80–84, 85–89, 90–94 and 95+.

### Birth cohort groups

Birth cohort was inferred using the year of investigation and the 5-year age groups (Appendix I in the Supporting Information, Figure S1). The birth years of the participants in different age groups were calculated using the formula in the succeeding text:


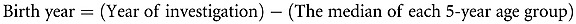


All the birth year data were categorised into five birth cohort groups: 1895 ∼ 1909, 1910 ∼ 1919, 1920 ∼ 1929, 1930 ∼ 1939 and 1940 ∼ 1950. The corresponding prevalence in the birth cohort groups was extracted, and the pooled prevalence of each group was estimated by meta-analysis.

### Median polish

Median polish is a method used to examine cohort effects in age-period contingency table (Keyes and Li, 2010). The additive effect of age and period was removed by iteratively subtracting the median value of each row and column. After several iterations, both row and column median is expected to approximate to zero (Selvin, 2004). If there is no cohort effect, the residuals, which can be considered as the potential effects of birth cohorts with error term, should be nearly zero after removing the effects of periods and age groups.

Because the studies that used older diagnostic criteria had more available data in the age groups 60–84 years, with more stable estimate of pooled prevalence, the analysis of median polish was only conducted for these age groups. Median residuals of the diagonals in age-period contingency table, which are regarded as different sets of birth cohorts, were calculated to present the change of residuals with birth cohorts.

### Estimating the pooled prevalence by age groups, periods and cohorts

The pooled estimates of five period groups were calculated by meta-analysis with a model adjusted for methodological factors and standardised to the 2010 China Census. Random-effect meta-analysis was used to calculate the pooled prevalence by age groups in different periods and birth cohorts based on estimates in the studies and their 95% binomial confidence intervals.

Estimates of dementia prevalence are known to be strongly influenced by diagnostic criteria (Erkingjuntti *et al*., 1997). Newer criteria, the Diagnostic and Statistical Manual of Mental Disorders, Fourth Edition (DSM-IV), 10/66 diagnostic algorithm (10/66) and Geriatric Mental State—Automated Geriatric Examination for Computer Assisted Taxonomy (GMS-AGECAT), are more likely to measure significantly higher prevalence than older criteria, such as Diagnostic and Statistical Manual of Mental Disorders, Third Edition (DSM-III), the International Classification of Diseases 10th (ICD-10), Chinese Classification of Mental Disorders (CCMD) and mixed diagnostic criteria (Wu *et al*., 2013). Although GMS-AGECAT is more equivalent to DSM-III-Revised (DSM-III-R), it was used as a newer diagnostic method in mainland China (Chen *et al*., 2012). The estimation of pooled prevalence was separated into two groups: older criteria (DSM-III, DSM-III-R, ICD-10, CCMD and mixed) and newer criteria (DSM-IV, DSM-IV-R, 10/66 and others).

### Sensitivity analysis

A sensitivity analysis was planned to explore whether cohort group classification methods might have affected the estimated prevalence. Because an individual's year of birth was generally unknown, the year of investigation and the median of age bands were used to calculate estimated birth years. This indicated the range of years that could represent the actual year of birth. The 5-year range of certain birth years could cross two 10-year cohort groups. For example, the range of 1929 (from 1927 to 1931) includes people born in both 1920 ∼ 1929 and 1930 ∼ 1939. Within any one age group, the proportion of younger people is usually higher than of older people: the age groups with the median estimated birth year 1929 are more likely to have more people born in 1929 ∼ 1931. Thus, it is arguably more appropriate to categorise their data into the 1930 ∼ 1939 birth cohort rather than 1920 ∼ 1929 as in the original analysis.

To examine the influence of these types of data, the sensitivity analysis was mainly conducted to the studies that used older criteria. The studies that contained the estimated birth year 1909, 1919, 1929, 1939 and 1949 were removed from the database. Dementia prevalence by age groups and birth cohorts was re-estimated by meta-analysis, and the results were compared with the previous analysis, which included all the available data.

## Results

Seventy-six prevalence studies of dementia in mainland China, Hong Kong and Taiwan were identified since 1980. Seventy studies that reported the age-stratified prevalence were included in the analysis. Forty-five studies used older criteria (DSM-III, DSM-III-R, ICD-10, CCMD and mixed), and 25 studies used newer criteria (DSM-IV, DSM-IV-R, 10/66 and GMS-AGECAT).

### The variations of prevalence in different periods

**Figure 1:**
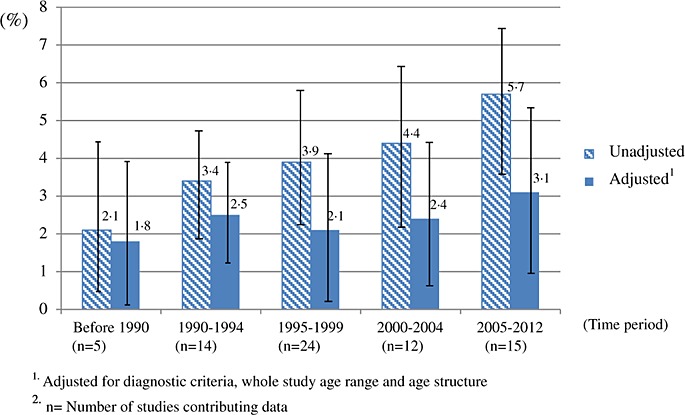
Prevalence of dementia in the population aged 60 years and over by different periods.

Most of the prevalence studies of dementia are concentrated in the 1990s. There were 38 studies in this period. Only five studies conducted before 1990 were identified. Twelve and 15 studies were conducted during the years 2000 ∼ 2004 and 2005 ∼ 2012, respectively. The unadjusted prevalence of dementia in mainland China, Hong Kong and Taiwan increased monotonically from 2.1% to 5.7% across the earliest to latest study periods with age standardisation using the 2010 China census population. After adjusting for diagnostic criteria, whole study age range and age structure, the prevalence of dementia in the older population aged 60 years and over fluctuated across time without significant differences, estimated as 1.8%, 2.5%, 2.1%, 2.4% and 3.1% for the five periods from pre-1990 to 2005 ∼ 2012 (Figure1). The estimates were adjusted to the population age 60 years and above and measured by older diagnostic criteria. It may appear that a slight increase was reported in the studies dating from 1995 to 2005, but statistically, the effect of period is uncertain. The change of diagnostic criteria explained a large amount of the increasing prevalence.

### Pooled prevalence of periods by 5-year age groups

**Figure 2.1:**
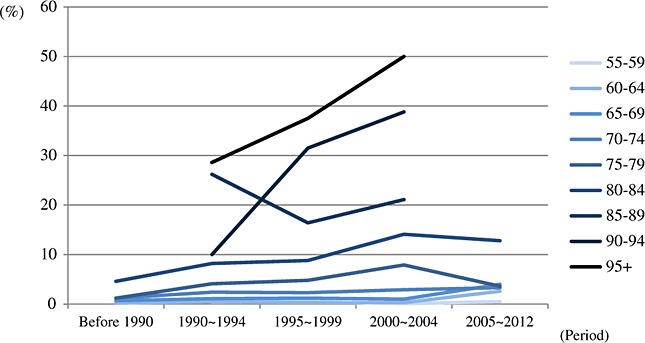
Pooled prevalence of dementia by period and age groups (older criteria group: Diagnostic and Statistical Manual of Mental Disorders, Third Edition (DSM-III), DSM-III-Revised, International Classification of Diseases 10th, Chinese Classification of Mental Disorders and mixed).

**Figure 2.2:**
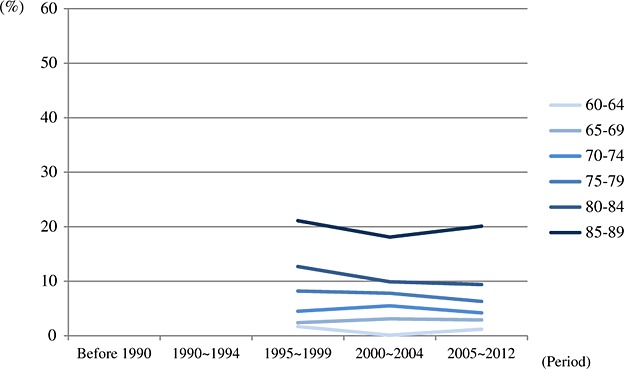
Pooled prevalence of dementia by period and age groups (newer criteria group: Diagnostic and Statistical Manual of Mental Disorders, Fourth Edition (DSM-IV), DSM-IV-Revised, 10/66 and Geriatric Mental State—Automated Geriatric Examination for Computer Assisted Taxonomy).

The pooled prevalence for period by age is presented in Figures 2.1 and 2.2. On the basis of diagnostic criteria used in earlier studies, there is a slight increase of the prevalence in the period of 2000 ∼ 2004. However, the numbers of studies contributing to these estimates were small (less than five). The pooled prevalence of the studies, which used newer criteria, had more consistent estimates across three periods from 1995 to 2012. The results of pooled prevalence by 5-year age groups were similar to previous section and indicated that there was no clear variation between different periods. More detailed information of pooled prevalence is provided in Appendix II in the Supporting Information.

### Median polish: investigating potential cohort effect

**Figure 3:**
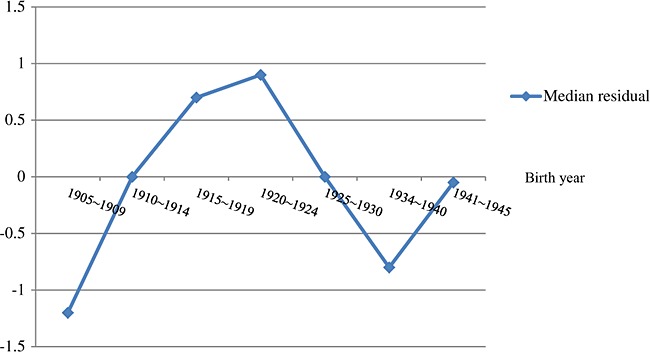
Median residuals in different birth cohorts.

After removing the effects of age and period using median polish, the median residuals by birth years are shown in Figure3, and detailed values are provided in Appendix III in the Supporting Information. The residual values were not close to zero and changed with different birth cohorts. This indicates potential cohort effects, suggesting that it is worth estimating pooled prevalence by age groups and birth cohorts.

### Pooled prevalence of birth cohorts by 5-year age groups

**Figure 4.1:**
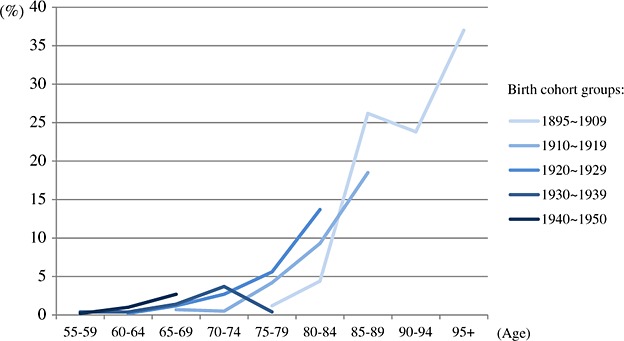
Age-stratified prevalence of dementia by 5-year birth cohorts (older criteria group: Diagnostic and Statistical Manual of Mental Disorders, Third Edition (DSM-III), DSM-III-Revised, International Classification of Diseases 10th, Chinese Classification of Mental Disorders and mixed).

**Figure 4.2:**
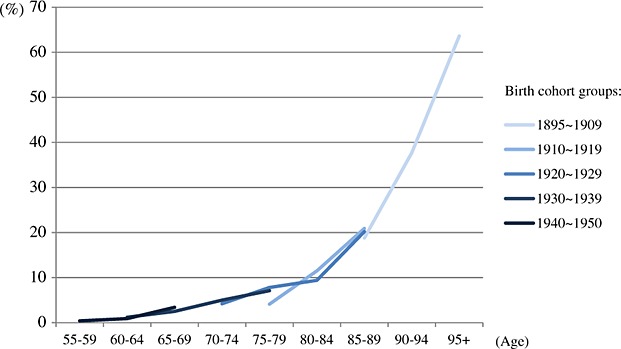
Age-stratified prevalence of dementia by 5-year birth cohorts (newer criteria group: Diagnostic and Statistical Manual of Mental Disorders, Fourth Edition (DSM-IV), DSM-IV-Revised, 10/66 and Geriatric Mental State—Automated Geriatric Examination for Computer Assisted Taxonomy).

Available data were categorised into the following birth cohort groups 1895 ∼ 1909, 1910 ∼ 1919, 1920 ∼ 1929, 1930 ∼ 1939 and 1940 ∼ 1950 (Table 1). Figure4.1 depicts the estimated prevalence of different birth cohorts by age groups among the studies that used earlier criteria. Higher pooled prevalence was found in recent cohorts in the same age group. The age-stratified prevalence gradually increased from less recent to recent birth cohorts, and there are larger differences in the age groups over 70 years. However, there is no clear difference between birth cohorts, which used newer diagnostic criteria. The pooled prevalence between various birth cohorts nearly overlapped in the same age groups, but the sample size for estimation was generally small (Figure4.2). More detailed information of pooled prevalence is provided in Appendix IV in the Supporting Information.

**Table:** The numbers of studies contributing to specific age/birth cohort cells

	N*	55–59	60–64	65–69	70–74	75–79	80–84	85–89	90–94	95+
1895 ∼ 1909	16/3					2/0	4/0	2/1	5/1	3/1
1910 ∼ 1919	37/10			3/0	5/0	11/1	14/6	4/3		
1920 ∼ 1929	40/25		4/0	11/0	14/9	8/10	3/4	0/2		
1930 ∼ 1939	21/29	1/0	8/5	8/11	3/8	1/5				
1940 ∼ 1950	11/14	5/4	5/4	1/6						

* Older/newer diagnostic criteria.

### Sensitivity analysis

Seven studies included the estimated birth year 1939, 1929, 1919 and 1909 (Li *et al*., 1989; Chen *et al*., 1992; Gao *et al*., 1994; Tang *et al*., 1998; Zhou *et al*., 2002; Tang *et al*., 2007; Chen *et al*., 2009). Five of them used older diagnostic criteria, and two used newer diagnostic criteria. Most of the uncertain data had the estimated years 1929 or 1919, and the estimates of cohort groups 1910 ∼ 1919 and 1920 ∼ 1929 were more likely to be affected in the sensitivity analysis. In general, after removing the data with the potential problem of misclassification, the results were similar to the original analysis, but more variation was found in the groups with smaller sample sizes. Detailed results are included in Appendix IV in the Supporting Information.

## Discussion

### Main findings

This study examined temporal variation of prevalence of dementia in mainland China, Hong Kong and Taiwan drawing on a systematic review of all relevant published studies. There was no significant variation across periods after controlling for methodological factors. A potential birth cohort effect was found in the major studies, which used older diagnostic criteria and prevalence generally increased from less recent to more recent cohort groups. In the studies that used new diagnostic criteria, there was no clear cohort effect on prevalence, but it might be related to smaller sample size and unstable estimates.

### Age, period and cohort effect

The main focus of this analysis is to explore temporal variation of dementia prevalence in mainland China, Hong Kong and Taiwan, where dramatic societal changes have taken place over the last hundred years. It is important to consider the effect of age, period and cohort on the prevalence of dementia over time, linking it to potential influence of societal changes.

1. Age effect: Life expectancy, which is associated with social and economic development, could have been affected by several historical events, such as wars and hunger. In China, it has extended dramatically in the 20th century (Appendix V in the Supporting Information). In this study, the influence of extended life expectancy on the change of age structure was controlled using age standardisation in the meta-regression model. The residual effect of age and longer life expectancy might contribute to the increasing population in higher-risk age of developing dementia given the reducing threats of competing cause of death.
2. Period effect: The prevalence of dementia did not significantly vary between periods after controlling for methodological factors and age structure. Despite longer life expectancy (Appendix V in the Supporting Information), ageing in the Chinese population has not yet had its maximum impact on the ages at which dementia is commonest. Older people with dementia in earlier decades might have had higher mortality rate and be more likely to die from pneumonia, diarrhoea and other acute diseases (Ineichen, 1998). This might balance the prevalence across different periods, explaining the results.
3. Cohort effect: After excluding the effects of age and periods, the residuals indicated a potential birth cohort effect. Although the different results in the two diagnostic criteria groups might be closely related to the evolving of diagnostic methods and cognitive assessment tools in the last decades, a pattern of increasing prevalence from less recent to more recent birth cohorts was found in the majority of studies using the older diagnostic criteria and providing more information across longer periods and birth cohorts. The small effect of age on the five birth cohorts could be explained by the fact that life expectancy at birth only extended slightly, approximately from 30 to 40 years old (Appendix V in the Supporting Information).

The possible cohort effect of dementia prevalence might indicate the potential influence of societal changes and individual life experience on cognitive function in later life (Appendix VI in the Supporting Information). The Great Famine (1958 ∼ 1961) and the Cultural Revolution (1966 ∼ 1976) could have led to malnutrition and deterioration of education in early life, affecting the development of cognitive function with severe impact on younger birth cohorts (Gordon, 1997; Hall and Hendrie, 2012). The recent economic growth in China could have caused substantial changes of lifestyle factors such as the consumption of meat intake, smoking and lack of physical activity with the increasing risk of chronic diseases (diabetes, stroke and hypertension) and dementia (Albanese *et al*., 2009; Larson *et al*., 2013). These factors might be more likely to affect younger cohorts, who were in their late youth or middle ages, and be importantly associated to this increasing pattern from less to more recent cohorts.

A stable or reduction of dementia prevalence in European countries over the last 20 years has been reported in recent studies with consistent study methods and representative sampling (Lobo *et al*., 2007; Matthews *et al*., 2013; Qiu *et al*., 2013). Despite potential residual influences of methodological factors in the meta-analysis, the difference of cohort effects between the two regions might indicate the potential effect of societal changes on mental health in later life. In the past few decades, the highly developed countries in Western Europe have had more wealth and stability, compared with East Asia, providing beneficial environments for population health. However, countries in East Asia have started to face serious burdens of chronic diseases, such as diabetes, hypertension and cardiovascular diseases, and extended life expectancy. Despite apparent stability of prevalence, in the next few decades, the prevalence of dementia in China is predicted to substantially increase, similarly to the pattern seen in Japan and South Korea in the past 5–8 years (Sekita *et al*., 2010; Kim *et al*., 2011; Ikejima *et al*., 2012).

### Limitations

The influence of social environment on prevalence of dementia can be decomposed into different factors related to dementia with complex effects of age, period cohort on individuals' mental health outcomes (Appendix VI in the Supporting Information). However, it is difficult to conduct complete age-period-cohort modelling and robust statistical tests because of limited information and considerable variations between the studies. The influence of these societal changes might be modified by individual factors, but the characteristics of study population could not be controlled in this analysis. As a meta-analysis, this study only summarises some descriptive results from the previous studies and provides the snapshots of dementia prevalence at different time points and potential pointers to the change of societal environment.

The small sample size of marginal age groups limits our ability to provide reliable estimates for the older and younger ranges of older age. Because many prevalence studies were conducted in the 1990s, limited data can be used to estimate the age-stratified prevalence in other periods. An approximate method was used to categorise the birth cohort groups, but the accurate birth years of individuals are unknown. The sensitivity analysis used to examine the influence of misclassification showed similar results in older age groups (70–74, 75–79 and 80–84 years) with stable estimates. To reduce publication bias, the results of literature search were compared with the reference list of earlier reviews. Data from proceedings of conference or government reports were obtained from the original authors (Zhang *et al*., 2012; Prince *et al*., 2013).

Although some of the Chinese populations in Hong Kong and Taiwan experienced similar historical events as those in mainland China (i.e. Japanese invasion, World War II and the Civil War), Hong Kong and Taiwan have experienced not only a rapid progress in economic development but also in political and social environments since the beginning of 20th century. Both Hong Kong and Taiwan averaged 10 years higher life expectancy than mainland China after 1920s and underwent economic development in 1960s (Appendix V in the Supporting Information). Moreover, both Hong Kong and Taiwan have very different colonial histories. Unfortunately, only seven studies were conducted in Hong Kong and Taiwan, and this restricts our ability to explore the spatial–temporal variation in this region.

### Future direction of study

One of the substantial challenges in this study is to garner sufficient data to allow full consideration of study designs and methods in different studies. To reduce the influence of methodological factors, conducting longitudinal studies with the same survey designs and methods over time can provide more comparable, consistent information to explore the variation of dementia prevalence in different periods and birth cohorts and examine the effect size of ageing, period and cohort.

The results of this study indicate potential effect of birth cohorts with detectable increasing pattern of prevalence from less to more recent birth cohorts after controlling for diagnostic criteria. Although the effect size might be small, the cross generational effect could be substantial. It will be important to study the prevalence of dementia with a life course approach, which examines the early life history of individuals and its effect on the future decisions, events and health conditions (Ben-Shlomo and Kuh, 2002). The change of societies in East Asia provides a natural experiment to study the association between historical events, individual life experiences and cognitive function in later life (Kleiman, 1996). Continuing systematic investigation will help provide insight into early and mid-life determinants of cognitive decline, important for the development of future public health initiatives.

**Key points**

- This study investigates the temporal variations of dementia prevalence in mainland China, Hong Kong and Taiwan and explores the potential influence of societal changes.
- The results provide insight into the possible impact of dementia on population health in different periods and cohorts, suggesting potential cohort effects. Societal changes might affect early life experiences across different generations with substantial impacts on cognition in older age.

## Supporting Information

Supporting info item
