# Supplementary material for: Period, birth cohort and prevalence of dementia in mainland China, Hong Kong and Taiwan: a meta-analysis
Source: Int J Geriatr Psychiatry. 2014 May 22;29(12):1212–20. doi: 10.1002/gps.4148 (PMC4552972; doi:10.1002/gps.4148)
Supplement: Supplementary file 1 — Supporting info item [file gps0029-1212-sd1.docx]

**Time period, birth cohort and prevalence of dementia in mainland China, Hong Kong and Taiwan: a meta-analysis**

**Appendix**

I. The inference of birth cohort

II. Pooled prevalence by time periods and age groups

III. Median polish and residual value

IV. Pooled prevalence by birth cohorts and age groups

V. Life expectancy at birth in mainland China, Hong Kong and Taiwan from 1895~2010

VI. Societal changes and the factors related to prevalence of dementia

**I. The inference of birth cohort**

**Figure S1 The inference of birth cohort**

55-59

60-64

57

1995

1938

Year of Investigation

Five-year age group

The median of age group

Birth year

62

1933

65-69

67

1928

…….

70-74

72

1923

Birth cohort group

Cohort 4: 1930~1939

Cohort 3: 1920~1929

**Prevalence study**

**II. Pooled prevalence by time periods and age groups**

**Table S2.1** Pooled prevalence (%) by periods and age groups (older criteria group: DSM-III, -III-R, ICD-10, CCMD and mixed)

|  | **55-59** | **60-64** | **65-69** | **70-74** | **75-79** | **80-84** | **85-89** | **90-94** | **95+** |
| --- | --- | --- | --- | --- | --- | --- | --- | --- | --- |
| **Before 1990** |  | 0.2 (0.0, 0.5)  (n=3) | 0.7 (0.2, 1.1) (n=3) | 1.2 (0.0, 3.1) (n=2) | 1.2 (0.3, 2.1) (n=2) | 4.6 (0.0, 9.3) (n=2) |  |  |  |
| **1991~1994** | 0.4 (0.2, 0.6) (n=1) | 0.5 (0.0, 1.1) (n=3) | 1.1 (0.6, 1.7) (n=8) | 2.4 (1.3, 3.4) (n=8) | 4.1 (2.7, 5.5) (n=8) | 8.2 (4.9, 11.6) (n=7) | 26.2 (0.0, 69.8)  (n=2) | 10.0 (4.2, 15.8)  (n=2) | 28.6 (3.7, 53.4) (n=1) |
| **1995~1999** | 0.5 (0.0, 1.0) (n=2) | 0.3 (0.1, 0.6) (n=5) | 1.2 (0.8, 1.6) (n=7) | 2.3 (1.2, 3.5) (n=7) | 4.8 (3.8, 5.7) (n=8) | 8.8 (6.2, 11.4) (n=8) | 16.4 (8.2, 24.5) (n=3) | 31.5 (15.3, 47.7)  (n=2) | 37.5 (4.0, 71.0) (n=1) |
| **2000~2004** | 0.1 (0.0, 0.3) (n=1) | 0.3 (0.2, 0.4) (n=4) | 1.0 (0.1, 1.8) (n=3) | 2.9 (0.1, 5.7) (n=2) | 7.9 (2.8, 13.0) (n=2) | 14.0 (6.4, 21.7) (n=2) | 21.1 (14.1, 28.1) (n=1) | 38.8 (24.5, 53.1) (n=1) | 50.0 (18.7, 81.3) (n=1) |
| **2005~2012** | 0.5 (0.0, 1.1) (n=1) | 2.6 (0.0, 5.5) (n=2) | 4.0 (1.6, 6.3) (n=2) | 3.3 (0.2, 6.5) (n=2) | 3.6 (0.0, 10.1) (n=2) | 12.8 (9.8, 15.9) (n=2) |  |  |  |

n: the number of studies contributing to age/ time period cells

**Table S2.2** Pooled prevalence (%) by periods and age groups (newer criteria group: DSM-IV, -IV-R, 10/66 and GMS-AGECAT)

|  | **55-59** | **60-64** | **65-69** | **70-74** | **75-79** | **80-84** | **85-89** | **90-94** | **95+** |
| --- | --- | --- | --- | --- | --- | --- | --- | --- | --- |
| **Before 1990** |  |  |  |  |  |  |  |  |  |
| **1990~1994** |  |  |  |  |  |  |  |  |  |
| **1995~1999** | 0.5 (0.2, 0.9)  (n=3) | 1.7 (0.7, 2.7)  (n=4) | 2.4 (1.5, 3.2)  (n=5) | 4.5 (2.7, 6.2)  (n=6) | 8.2 (5.3, 11.2)  (n=6) | 12.7 (8.7, 16.8)  (n=4) | 21.1 (15.5, 26.7)  (n=3) |  |  |
| **2000~2004** |  | 0.1 (0.0, 0.61)  (n=1) | 3.1 (1.5, 4.8)  (n=5) | 5.5 (3.4, 7.6)  (n=5) | 7.8 (5.1, 10.5)  (n=4) | 9.9 (7.4, 12.4)  (n=2) | 18.1 (12.5, 24.8)  (n=1) | 37.7 (24.1, 51.4)  (n=1) | 63.6 (30.8, 89.1)  (n=1) |
| **2005~2012** |  | 1.2 (0.3, 2.1)  (n=3) | 2.9 (1.7, 4.1)  (n=7) | 4.2 (2.7, 5.7)  (n=6) | 6.3 (3.9, 8.6)  (n=6) | 9.4 (5.5, 13.3)  (n=4) | 20.2 (12.3, 28.1)  (n=2) |  |  |

n: the number of studies contributing to age/ time period cells

**III. Median polish and residual value**

**Table S3.1** The residuals after removing the effect of row (period) and column (age)

|  | **60-64** | **65-69** | **70-74** | **75-79** | **80-84** |
| --- | --- | --- | --- | --- | --- |
| **Before 1990** | 0.9 | 0.6 | 0.0 | -1.7 | -3.1 |
| **1990~1994** | 0.0 | -0.2 | 0.0 | 0.0 | -0.7 |
| **1995~1999** | -0.1 | 0.0 | 0.0 | 0.8 | 0.0 |
| **2000~2004** | -0.7 | -0.8 | 0.0 | 3.3 | 4.6 |
| **2005~2012** | 0.0 | 0.6 | -1.2 | -2.6 | 1.8 |

**Table S3.2** The residuals presented by different birth cohorts

| **Birth year** | **60-64** | **65-69** | **70-74** | **75-79** | **80-84** | **Median** |
| --- | --- | --- | --- | --- | --- | --- |
| **1905~1909** |  |  |  | -1.7 | -0.7 | -1.2 |
| **1910~1914** |  |  | 0.0 | 0.0 | 0.0 | 0.0 |
| **1915~1919** |  | 0.6 | 0.0 | 0.8 | 4.6 | 0.7 |
| **1920~1924** | 0.9 | -0.2 | 0.0 | 3.3 | 1.8 | 0.9 |
| **1925~1930** | 0.0 | 0.0 | 0.0 | -2.6 |  | 0.0 |
| **1934~1940** | -0.1 | -0.8 | -1.2 |  |  | -0.8 |
| **1941~1945** | -0.7 | 0.6 |  |  |  | -0.1 |

**IV. Poole prevalence by birth cohorts and age groups**

**Table S4.1** The pooled prevalence (%) by age and birth cohort groups (older criteria group: DSM-III, -III-R, ICD-10, CCMD and mixed)

|  | **55-59** | **60-64** | **65-69** | **70-74** | **75-79** | **80-84** | **85-89** | **90-94** | **95+** |
| --- | --- | --- | --- | --- | --- | --- | --- | --- | --- |
| **1895~1909** |  |  |  |  | 1.2 (0.3, 2.1)  (n=2) | 4.4 (2.8, 6.0)  (n=4) | 26.2 (0.0, 69.8)  (n=2) | 23.8 (7.9, 39.6)  (n=5) | 37.0 (20.2, 53.9)  (n=3) |
| **1910~1919** |  |  | 0.7 (0.2, 1.1)  (n=3) | 0.5 (0.1, 0.8)  (n=5) | 4.2 (3.0, 5.4)  (n=11) | 9.3 (7.3, 11.7)  (n=14) | 18.5 (12.9, 24.0)  (n=4) |  |  |
| **1920~1929** |  | 0.2 (0.0, 0.3)  (n=4) | 1.2 (0.7, 1.7)  (n=11) | 2.7 (1.9, 3.6)  (n=14) | 5.6 (4.5, 6.7)  (n=8) | 13.7 (10.5, 16.9)  (n=3) |  |  |  |
| **1930~1939** | 0.4 (0.2, 0.6)  (n=1) | 0.4 (0.2, 0.6)  (n=8) | 1.4 (0.7, 2.1)  (n=8) | 3.7 (1.7, 5.8)  (n=3) | 0.4 (0.0, 2.4)  (n=1) |  |  |  |  |
| **1940~1950** | 0.2 (0.0, 0.4)  (n=5) | 1.0 (0.4, 1.7)  (n=5) | 2.7 (0.7, 4.8)  (n=1) |  |  |  |  |  |  |

n: the number of studies contributing to age/ birth cohot cells

**Table S4.2** Pooled prevalence (%) by birth cohorts and age groups (newer criteria group: DSM-IV, -IV-R, 10/66 and GMS-AGECAT)

|  | **55-59** | **60-64** | **65-69** | **70-74** | **75-79** | **80-84** | **85-89** | **90-94** | **95+** |
| --- | --- | --- | --- | --- | --- | --- | --- | --- | --- |
| **1895~1909** |  |  |  |  |  |  | 18.8 (13.1, 25.4)  (n=1) | 37.7 (24.8, 52.1)  (n=1) | 63.6 (30.8, 89.1)  (n=1) |
| **1910~1919** |  |  |  |  | 4.1 (2.2, 7.2)  (n=1) | 11.5 (9.1, 14.0)  (n=6) | 20.9 (15.2, 26.6)  (n=3) |  |  |
| **1920~1929** |  |  |  | 4.2 (3.1, 5.4)  (n=9) | 7.8 (5.7, 9.9)  (n=10) | 9.4 (5.5, 13.3)  (n=4) | 20.2 (12.3, 28.1)  (n=2) |  |  |
| **1930~1939** |  | 1.2 (0.5, 2.0)  (n=5) | 2.5 (1.8, 3.2)  (n=11) | 5.0 (3.5, 6.6)  (n=8) | 7.1 (5.0, 9.2)  (n=5) |  |  |  |  |
| **1940~1950** | 0.4 (0.1, 0.7)  (n=4) | 0.9 (0.1, 1.7)  (n=4) | 3.4 (2.0, 4.9)  (n=6) |  |  |  |  |  |  |

n: the number of studies contributing to age/ time period cells

**Table S4.3** Comparison of original and sensitivity analysis: the pooled prevalence (%) by age and birth cohort groups (older criteria group: DSM-III, -III-R, ICD-10, CCMD and mixed)

|  | **60-64** |  | **65-69** |  | **70-74** |  | **75-79** |  | **80-84** |  |
| --- | --- | --- | --- | --- | --- | --- | --- | --- | --- | --- |
|  | **Original** | **Sensitivity analysis** | **Original** | **Sensitivity analysis** | **Original** | **Sensitivity analysis** | **Original** | **Sensitivity analysis** | **Original** | **Sensitivity analysis** |
| **1895~1909** |  |  |  |  |  |  | 1.2 (0.3, 2.1)  (n=2) |  | 4.4 (2.8, 6.0)  (n=4) | 4.9 (1.3, 12.0)  (n=1) |
| **1910~1919** |  |  | 0.7 (0.2, 1.1)  (n=3) | 1.9 (0.6, 4.5)  (n=1) | 0.5 (0.1, 0.8)  (n=5) | 1.1 (0.0, 3.4)  (n=2) | 4.2 (3.0, 5.4)  (n=11) | 4.7 (3.3, 6.0)  (n=9) | 9.3 (7.3, 11.7)  (n=14) | 9.2 (6.8, 11.6)  (n=13) |
| **1920~1929** | 0.2 (0.0, 0.3)  (n=4) | 1.1 (0.2, 3.2)  (n=1) | 1.2 (0.7, 1.7)  (n=11) | 1.3 (0.8, 1.9)  (n=9) | 2.7 (1.9, 3.6)  (n=14) | 2.6 (1.8, 3.5)  (n=13) | 5.6 (4.5, 6.7)  (n=8) | 5.4 (4.2, 6.6)  (n=7) | 13.7 (10.5, 16.9)  (n=3) | 15.5 (11.2, 19.8)  (n=2) |
| **1930~1939** | 0.4 (0.2, 0.6)  (n=8) | 0.4 (0.2, 0.6)  (n=7) | 1.4 (0.7, 2.1)  (n=8) | 0.9 (0.5, 1.3)  (n=7) | 3.7 (1.7, 5.8)  (n=3) | 3.1 (0.4, 5.8)  (n=2) | 0.4 (0.0, 2.4)  (n=1) | 0.4 (0.0, 2.4)  (n=1) |  |  |
| **1940~1950** | 1.0 (0.4, 1.7)  (n=5) | 1.0 (0.4, 1.7)  (n=5) | 2.7 (0.7, 4.8)  (n=1) | 2.7 (0.7, 4.8)  (n=1) |  |  |  |  |  |  |

Shading: The estimated prevalence which was affected in the sensitivity analysis, which removed the studies with the estimated birth year 1949, 1939, 1929, 1919, and 1909.

**V. Life expectancy at birth in mainland China, Hong Kong and Taiwan from 1895 to 2010**

The division of People’s Republic China and Republic of China (Taiwan)

Taiwan: Japanese colonial period

Hong Kong: British colonial period

(Except 1941~1945, Japanese occupation in World War II)

The end of British colonial period

Cultural Revolution

Year

Mainland China:

China Empire

The end of Japanese colonial period

Life expectancy (year old)

Foundation of Republic of China

Japan invasion & World War II

Great Famine

(Source: Gapminder)

**VI. Societal changes and the factors related to prevalence of dementia**

Cohort effect

Age effect

Period effect

**The change of social environment**

- Economic development

- Wars and social disruption

- Political conflicts and influence

**Prevalence of dementia**

in different time periods and birth cohorts

-Life expectancy and age structure of population

-Chronic diseases

Lack of education

Negative life experience -Hunger, poverty

-Stress

-Poor quality of life

Concepts to mental illnesses

Characteristics of study population, individual risk factors

Research interests, varying tools for assessment
